# Prevalence and risk factors of paradoxical tuberculosis associated immune reconstitution inflammatory syndrome among HIV-infected patients in Beijing, China

**DOI:** 10.1186/s12879-020-05225-x

**Published:** 2020-07-31

**Authors:** Ming Xue, Ruming Xie, Yu Pang, Shuo Yan, Yanni Du, Chunshuang Guan, Budong Chen

**Affiliations:** 1 Department of Radiology, Beijing Ditan Hospital, Capital Medical University, No. 8, Jingshun East Street, Chaoyang District, Beijing, 100015 P. R. China; 2 National Clinical Laboratory on Tuberculosis, Beijing Chest Hospital, Capital Medical University/Beijing Tuberculosis & Thoracic Tumor Research Institute, Beijing, 101149 P. R. China

**Keywords:** Tuberculosis, Immune reconstitution inflammatory syndrome, Paradoxical

## Abstract

**Background:**

In this study, we aimed to describe the prevalence, clinical presentation and risk factors of paradoxical tuberculosis-associated immune reconstitution inflammatory syndrome (TB-IRIS) cases in China.

**Methods:**

We performed a descriptive analysis of demographic and clinical data of HIV/TB coinfected patients receiving ART at Beijing Ditan Hospital between January 2014 and October 2018.

**Results:**

Of 199 patients included, 45 (22.6%) developed paradoxical TB-IRIS, and 19 (9.5%) TB-IRIS cases presented miliary TB. The pre-ART CD4 count lower than 50 cells/mm^3^ was found to be significantly associated with development of TB-IRIS. Similarly, patients with higher than 4-fold increase in CD4 cell count after antiretroviral therapy (ART) had significantly higher odds of having TB-IRIS. When patients aged 25–44 years were utilized as the control group, youths (< 25 years old) were more likely to have miliary TB. No significant difference was observed in the intervals from initiation of ART to IRIS presentation between miliary and non-miliary group.

**Conclusions:**

In conclusion, our data demonstrate that approximate one quarter of patients coinfected with TB and HIV develop paradoxical TB-IRIS after initial of ART therapy in China. Lower baseline CD4 count and rapid increase in CD4 count are the major risk factors associated with the occurrence of paradoxical TB-IRIS.

## Background

Tuberculosis (TB) remains the most common opportunistic pathogen in human immunodeficiency virus (HIV)-infected patients [1, 2] As the leading causes of death among people with HIV, an estimated 0.3 million HIV-infected cases died from TB in 2017 [2]. Antiretroviral therapy (ART) is an essential intervention to improve survival in patients with advanced HIV/TB [3] However, this strategy also increase risk for worsening tuberculosis symptoms or development of new tuberculosis symptoms despite virological efficacy. This clinical condition is known as TB-associated immune reconstitution inflammatory syndrome (TB-IRIS) [3]. It often emerges shortly after ART initiation, and is characterized by a transient but sometimes severe local and systemic inflammatory response [1], majorly reflecting an exaggerated immune response to *Mycobacterium tuberculosis* antigens during the reconstitution of compromised immune system [4, 5].

IRIS is characterized by a transient but sometimes severe local and systemic inflammatory response directed against a known condition (e.g., opportunistic pathogens or autoimmune diseases) in HIV-1 infected patients shortly after ART initiation. In HIV-infected patients, rates of TB-IRIS range between 8 and 54%, and the occurrence of this severe form of TB is associated with dramatically increased mortality. With the proposed widespread use of early ART in HIV/TB coinfected patients, the incidence of TB-IRIS will likely rise [6].

Although the underlying immunological mechanisms of TB-RIIS are incompletely understood, a few clinical studies have demonstrated that risk factors for the development of TB-IRIS include low pre-ART CD4+ T cell counts, stronger CD4+ T cell increase after ART and short interval between starting antituberculous therapy and ART [7, 8], while the other studies did not find the relationship between these risk factors and developing IRIS [9]. These contradictory results indicate that the small sample size of previous studies may negatively affect the generalization of these conclusions among different populations.

China is currently undergoing a serious TB epidemic, with an estimated 0.89 million of incidence cases in 2017 [2]. In 2018, there were an estimated 18,000 TB patients and 2400 TB deaths among HIV-positive people in this country [2]. An alarming public health threat has emerged with the concomitant epidemics of HIV and TB in past decades, especially in sex workers, intravenous drug users, and former plasma donors, thereby directly leading to an increasing incidence of TB-IRIS. To date, no specific diagnostic test is available to confirm IRIS diagnosis. Thus, evaluating the risk factors associated with developing IRIS could be an alternative to identify patients at risk for developing TB-IRIS. Unfortunately, little is known about incidence and clinical risk factors of TB-IRIS among patients initiating ART in China. To address this concern, we aimed to describe the prevalence, clinical presentation and risk factors of paradoxical TB-IRIS cases in China. A secondary objective was to determine factors associated with an increased risk of miliary TB among paradoxical TB-IRIS cases.

## Methods

### Patients

We performed a retrospective analysis of demographic and clinical data of HIV/TB coinfected patients receiving ART at Beijing Ditan Hospital between January 2014 and October 2018. Beijing Ditan Hospital, designated a National Quality Control Centre on Infectious Diseases, is a 1158-bed general hospital that delivers specialized treatment for patients infected with HIV, hepatitis virus and other infectious diseases, except for TB, whereas the patients coinfected with HIV and TB were referred to this hospital for treatment of TB and HIV meanwhile. This hospital provides a tertiary care service for HIV-infected patients from Beijing Municipality and surrounding regions. Patients were clinically assessed, and had a computerized tomography (CT) scan, routine blood counts, biochemical tests, urinalysis, CD4 count, HIV RNA load and tuberculosis-specific interferon-gamma release assay (IGRA) at initial visit. After the primary treatment, radiological examination and routine laboratory tests were performed at completion of 2, 4, 8, 16 weeks, and then every 3 months of treatment, or after onset of progressively clinical TB symptoms for patients coinfected with HIV/TB. The study inclusion criteria were: i. adult aged ≥18 years; ii. confirmed HIV infection; iii. Patients receiving ART; iv. patients with positive tuberculosis-specific IGRA test irrespective of clinical presentation of TB disease. Exclusion criteria were: i. pregnant patients; ii. those on immunomodulatory agents; iii. Patients infected with RIF-resistant tuberculosis.

### Case definitions

Bacteriogically confirmed TB cases were defined as disease proven by isolation of *Mycobacterium tuberculosis*, and/or positive histopathological evidence. Clinically diagnosed TB cases were defined as those with a suggestive chest radiograph, having clinical symptoms together with an appropriate response to anti-TB treatment. Paradoxical TB-IRIS was defined as worsening clinical tuberculosis symptoms and/or worsening radiological features of tuberculosis at any time after ART initiation. Each suspected case of paradoxical TB-IRIS reviewed by two members of the clinical coordination team. Miliary tuberculosis was defined as the presence of military nodules on thoracic radiologic imaging in patients who presented with clinical symptoms suggestive of tuberculosis such as prolonged fever, night sweats, anorexia and weight loss.

### Data collection and analysis

Demographic and clinical data were collected from electronic patient record system, including gender, age group, CD4 count and HIV RNA load. For patients with TB-IRIS, CD4 count and HIV RNA load were obtained at baseline and at the onset of TB-IRIS, respectively. For patients without TB-IRIS, these laboratory data were obtained at baseline and 3 months post-ART initiation, respectively. The patients were classified into three groups on the basis of baseline CD4 T lymphocyte cell levels as previously described [10]. In primary univariate logistic regression, patients with TB-IRIS were compared to patients without TB-IRIS as controls. Multivariable logistic regression models were built by using forward stepwise logistic regression procedures with the inclusion of variables with *P* < 0.1 in the univariate analysis. The difference was declared as significant if a *P* value was less than 0.05. All statistical analyses were performed using SPSS 20.0 software (SPSS Inc., Chicago, Illinois, USA).

## Results

### Patient enrolment

**Fig. 1:**
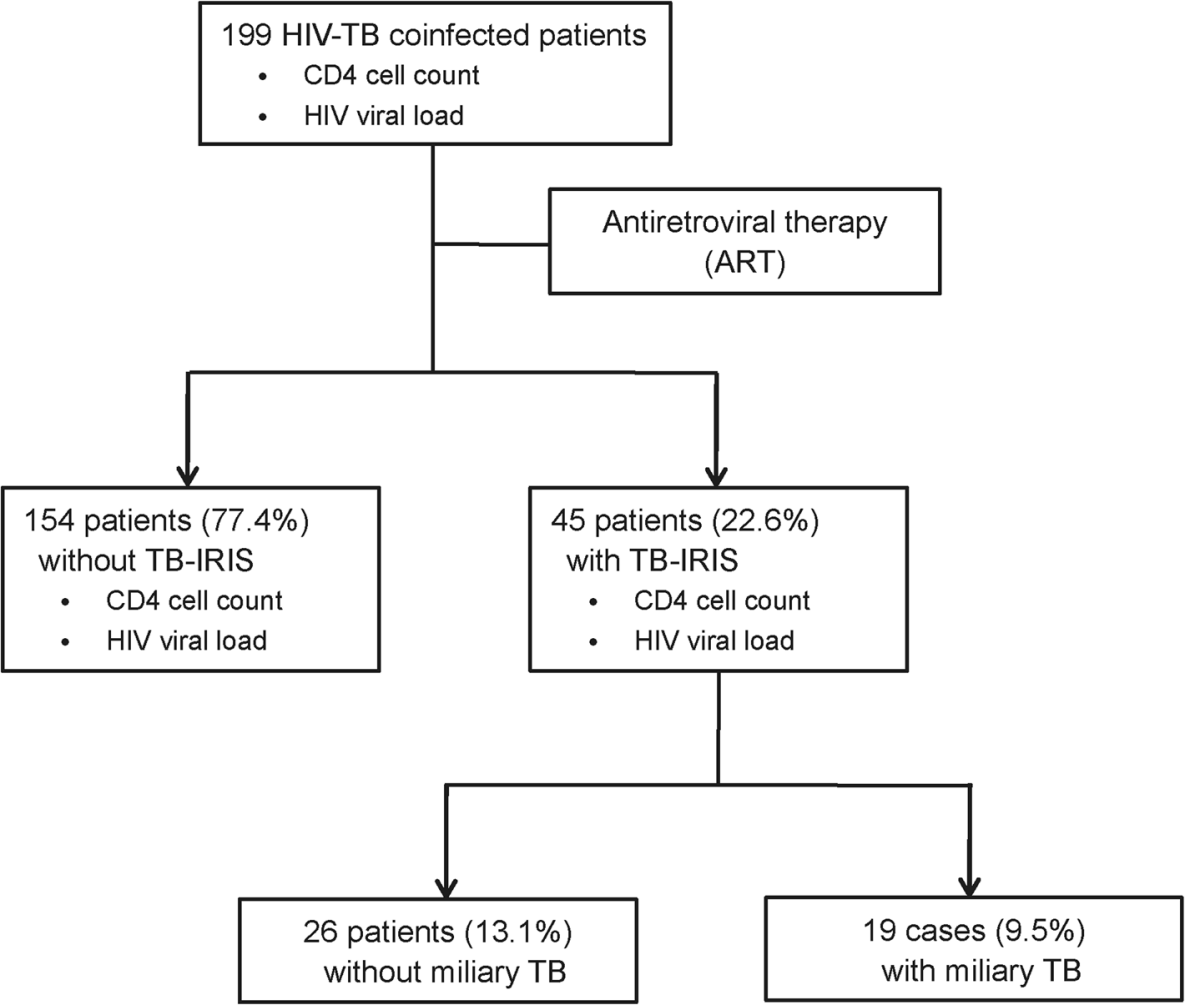
Enrolment of patients

Between January 2014 and October 2018, a total of 199 patients coinfected with TB and HIV, who were given combination antiretroviral therapy, were included in this study (Fig. 1). 174 (87.4%, 174/199) started a combination of tenofovir, lamivudine and efavirenz, while the other 25(12.6%, 25/199) were treated with a combination of tenofovir, lamivudine and lopinavir/ritonavir. Of these 199 patients, 45 (22.6%, 45/199) developed paradoxical TB-IRIS. In addition, 19 (9.5%, 19/199) TB-IRIS cases presented miliary TB.

### Risk factors of TB-IRIS

In the multivariate analysis, the patients aged 45–65 years had lower odds of developing TB-IRIS than patients aged 25–44 years [adjusted odds ratio (aOR): 0.355, 95% confidence interval (CI): 0.127–0.993]. Similarly, patients with higher than 4-fold increase in CD4 cell count after ART had significantly higher odds of having TB-IRIS (aOR: 2.614, 95% CI: 1.288–5.303) (Table 1).

**Table 1:** Multivariate analysis of the characteristics associated with the presence of TB-IRIS

Characteristics	TB-IRIS (45)	Non-TB-IRIS (154)	Total (199)	Crude OR^**b**^	***P*** value	Adjusted OR^**c**^	***P*** value
	N (%)	N (%)	N (%)	(95% CI)		(95% CI)	
**Gender**							
Male	42 (93.3)	146 (94.8)	188 (94.5)	1	Ref.		
Female	3 (6.7)	8 (5.2)	11 (5.5)	1.304 (0.331–5.133)	0.705		
**Age group (years)**							
< 25	9 (20.0)	16 (10.4)	25 (12.6)	1.744 (0.699–4.351)	0.233	2.024 (0.786–5.212)	0.144
25–44	30 (66.7)	93 (60.4)	123 (61.8)	1	Ref.	1	Ref.
45–64	5 (11.1)	42 (27.3)	47 (23.6)	0.369 (0.134–1.018)	0.054	0.355 (0.127–0.993)	0.049
> 64	1 (2.2)	3 (1.9)	4 (2.0)	1.033 (0.104–10.310)	0.978	1.603 (0.156–16.479)	0.692
**Initial CD4 cell count (cells/mm**^**3**^**)**							
> 100	2 (4.4)	24 (15.6)	26 (13.1)	1	Ref.		
50–100	3 (6.7)	25 (16.2)	28 (14.1)	1.440 (0.221–9.388)	0.703		
< 50	40 (88.9)	105 (68.2)	145 (72.9)	4.571 (1.033–20.238)	0.045		
**CD4 cell count after ART (cells/mm**^**3**^**)**							
> 100	21 (46.7)	71 (46.1)	92 (46.2)	1	Ref.		
50–100	16 (35.6)	42 (27.3)	58 (29.1)	1.288 (0.606–2.738)	0.511		
< 50	8 (17.8)	41 (26.6)	49 (24.6)	0.660 (0.268–1.623)	0.365		
**Increase in CD4 cell count**							
< 4 folds	21 (46.7)	103 (66.9)	124 (62.3)	1	Ref.	1	Ref.
≥ 4 folds	24 (53.3)	51 (33.1)	75 (37.7)	2.308 (1.175–4.533)	0.015	2.614 (1.288–5.303)	0.008
**HIV virial load (copies/mL)**							
≤ 1000 copies	24 (53.3)	94 (61.0)	118 (59.3)	1	Ref.		
> 1000 copies	21 (46.7)	60 (39.0)	81 (40.7)	1.371 (0.702–2.677)	0.356		
**HAART regimen**^a^							
First-line	38 (84.4)	136 (88.3)	174 (87.4)	1	Ref.		
Second-line	7 (15.6)	18 (11.7)	25 (12.6)	1.392 (0.541–3.578)	0.493		

^a^First-line regimen includes tenofovir, lamivudine and efavirenz; Second-line regimen includes tenofovir, lamivudine and lopinavir/ritonavir

^b^OR, odds ratio; CI, confidential interval

^c^The initial CD4 cell count is considered as a confounding variable of increase in CD4 cell count, which is removed in the multivariate analysis

### Risk factors of miliary TB among TB-IRIS cases

**Fig. 2:**
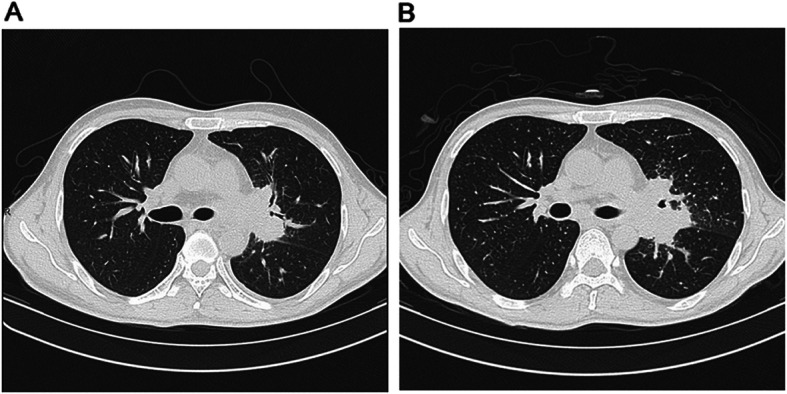
Contrast-enhanced CT images of an TB-IRIS patient experiencing paradoxical TB-IRIS. **a**. A man was diagnosed as lymphatic tuberculosis at the time of HIV diagnosis. No obvious abnormal findings were recorded in the lung fields. **b**. ART was intiated after 14 days of anti-TB treatment. After 28 days of ART, he had worsening lymph node enlargement and military infiltration of the lungs

A total of 19 patients met the criteria for miliary TB. As shown in Fig. 2, a man was diagnosed as lymphatic tuberculosis at the time of HIV diagnosis. No obvious abnormal findings were recorded in the lung fields. After 28 days of ART, he had worsening lymph node enlargement and military infiltration of the lungs. We further analysed the risk factors of miliary TB among TB-IRIS cases. The distribution of miliary TB among TB-IRIS cases differed among different age groups. When patients aged 25–44 years were utilized as the control group, youths (< 25 years old) were more likely to have miliary TB (OR: 8.167, 95% CI: 1.412–47.221). In contrast, sex, pre-ART CD4 count and HIV virial load had no significant influence on the prevalence of miliary TB (*P* > 0.05) (Table 2).

**Table 2:** Univariate analysis of the characteristics associated with miliary TB among TB-IRIS

Characteristics	Miliary TB (19) N (%)	Non-miliary TB (26) N (%)	Total (45) N (%)	Crude Or^**b**^ (95% CI)	***P*** value
**Gender**					
Male	18 (94.7)	24 (92.3)	42 (93.3)	1	Ref.
Female	1 (5.3)	2 (7.7)	3 (6.7)	0.667 (0.056–7.937)	0.748
**Age group (years)**					
< 25	7 (36.8)	2 (7.7)	9 (20.0)	8.167 (1.412–47.221)	0.019
25–44	9 (47.4)	21 (80.8)	30 (66.7)	1	Ref.
45–64	3 (15.8)	2 (7.7)	5 (11.1)	3.500 (0.497–24.654)	0.208
> 64	0 (0.0)	1 (3.8)	1 (2.2)	–	1
**Initial CD4 cell count**					
> 100	1 (5.3)	1 (3.8)	2 (4.4)	1	Ref.
50–100	1 (5.3)	2 (7.7)	3 (6.7)	0.500 (0.013–19.562)	0.711
< 50	17 (89.5)	23 (88.5)	40 (88.9)	0.739 (0.043–12.674)	0.835
**CD4 cell count after ART**					
> 100	6 (31.6)	15 (57.7)	21 (46.7)	1	Ref.
50–100	8 (42.1)	8 (30.8)	16 (35.5)	2.500 (0.640–9.766)	0.188
< 50	5 (26.3)	3 (11.5)	8 (17.8)	4.167 (0.749–23.179)	0.103
**Increase in CD4 cell count**					
< 4 folds	9 (47.4)	12 (46.2)	21 (46.7)	1	Ref.
≥ 4 folds	10 (52.6)	14 (53.8)	24 (53.3)	0.952 (0.291–3.117)	0.936
**HIV virial load**					
≤ 1000 copies	10 (52.6)	14 (53.8)	24 (53.3)	1	Ref.
> 1000 copies	9 (47.4)	12 (46.2)	21 (46.7)	1.050 (0.321–3.436)	0.936
**HAART regimen**^**a**^					
First-line	15 (78.9)	23 (88.5)	38 (84.4)	1	Ref.
Second-line	4 (21.1)	3 (11.5)	7 (15.6)	2.044 (0.400–10.457)	0.390

^a^First-line regimen includes tenofovir, lamivudine and efavirenz; Second-line regimen includes tenofovir, lamivudine and lopinavir/ritonavir

^b^OR, odds ratio; CI, confidential interval

### Intervals between ART and IRIS diagnosis

**Fig. 3:**
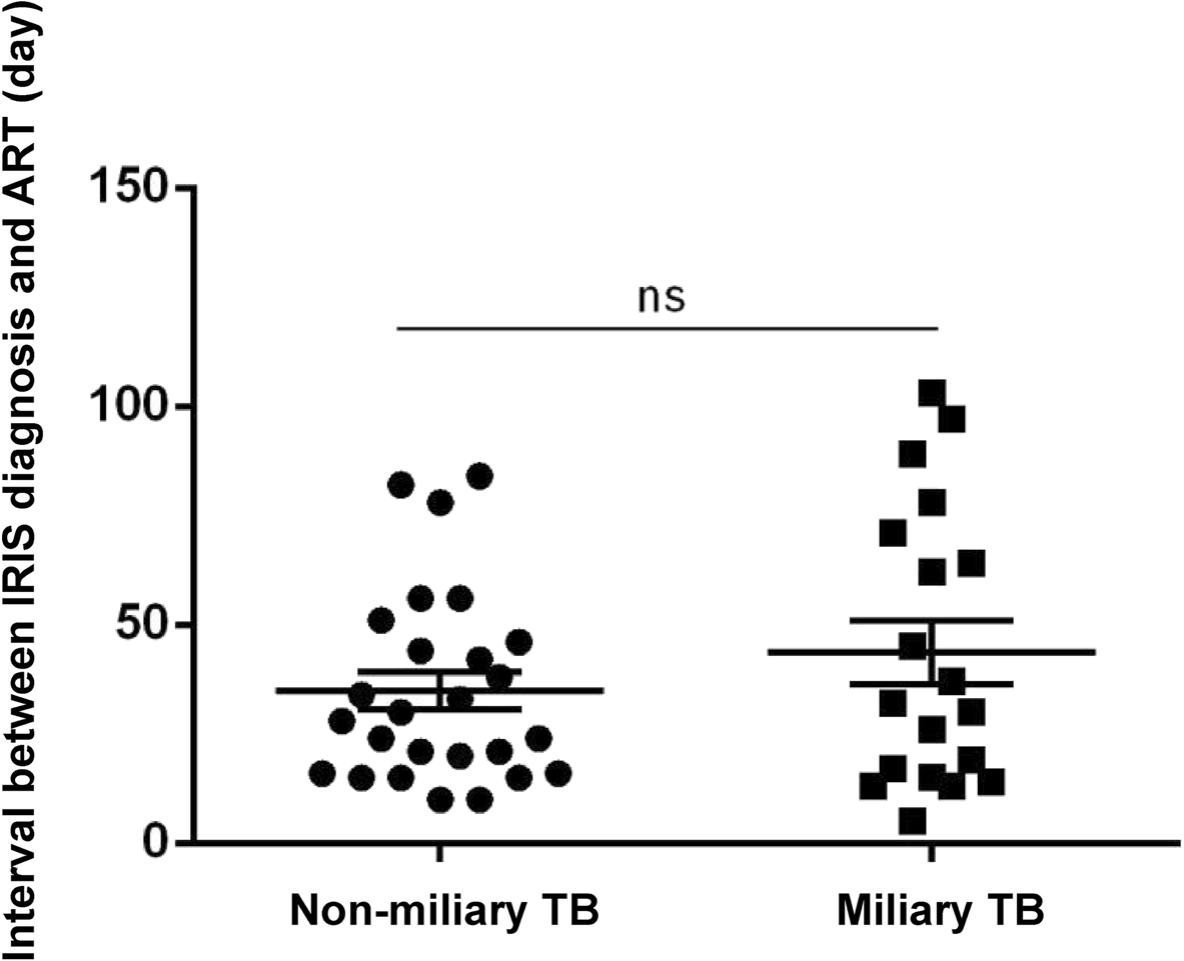
Intervals between TB-IRIS diagnosis and the initial of ART. The mean intervals for non-miliary group and miliary group are 35.0 ± 4.3 days and 43.7 ± 7.3 days, respectively. No significant difference is noted between two group (*P* = 0.08)

The intervals from initiation of ART to IRIS presentation were compared between miliary group and non-miliary group. The mean interval for patients in miliary group was 43.7 ± 7.3 days, ranging from 5 to 103 days, while that for patients in non-miliary group was 35.0 ± 4.3 days, ranging from 10 to 84 days. There was no significant difference between two groups (*P* = 0.08) (Fig. 3).

## Discussion

To our best knowledge, this is the first study to report the prevalence and risk factors of paradoxical TB-IRIS in China. Our data demonstrate that 22.6% of patients coinfected with HIV and TB develop paradoxical TB-IRIS in the Chinese population. The prevalence of paradoxical TB-IRIS is similar to that in Ethiopia (22.4%) [11] and that in Spain (24.3%) [12], although it is lower than that in USA (36.4%) [13] and that in Croatia (40.7%) [14], and higher than that in India (7.6%) [15] and that in South Africa (11.2%) [16]. A recent meta-analysis reveals that the pooled prevalence in Asian studies (15%) is significantly lower than that in European studies (33%) [17], which is also lower than our result. On one hand, the diversity in prevalence may be partly explained by geographic region and study design [17]. In most of studies from Europe, the less stringent criteria were used to define TB-IRIS, thus the ascertainment bias related to study design may be a major explanation for the differences observed across studies [17]. On the other hand, we hypothesize that the combination of unsatisfactory quality and limited access to health care may be contributed to the missed diagnosis of paradoxical TB-IRIS, thus resulting in underestimation of its incidence in Asia.

The major risk factors that are reported to increase the risk of paradoxical TB-IRIS include low baseline CD4 count and high baseline load [9, 18, 19] In consistent to previous studies, our report also identified an association between the occurrence of TB-IRIS and the pre-ART CD4 count lower than 50 cells/mm^3^. In addition, we found that a greater increase in CD4 count presented a risk factor for the development of paradoxical TB-IRIS. Similar to our observation, a clinical trial by Breton and colleagues reported that IRIS was associated with increases in the CD4 cell percentage after 1 month of antiretroviral therapy [20]. The change in CD4 count during early ART reflects rapid immunological recovery among HIV-infected cases [17]. Although an effective CD4 T cell response is essential to limit tubercle bacilli throughout the body, it can also accelerate the development of progressively destructive lesions in the lesions [21]. The rapid increase in CD4 cell count provides better protection against opportunistic infections in the HIV-infected persons. However, the activation of CD4 cell response may represent a two-edged sword, with a damage on the host mediated by an excessive or inappropriate immune response [22]. Thus, our results highlight the crucial importance of balance between protection and immunopathology during the early ART therapy. The moderate reconstruction of the immune system will decrease the risk of paradoxical TB-IRIS via monitoring CD4 T cells.

Miliary TB is a potentially lethal form of tuberculosis resulting from haematogenous dissemination of *M. tuberculosis*, accounting for 1–2% of patients with tuberculosis [23]. In this study, more than 40% of paradoxical TB-IRIS cases developed miliary TB after initial ART therapy. The extremely high incidence of miliary TB supports the previous reports that the risk of developing military TB is the greatest in patients with altered host immunity, including HIV-infected patients and individuals with immunosuppressive and immunomodulator drugs [24]. In addition, we also found that patients younger than 25 years of age were most likely to have miliary TB among paradoxical TB-IRIS cases. In consistent to our observation, Rieder et al. found that patients younger than 15 or older than 65 years of age had significantly higher odds of having miliary TB [25]. The difference remains largely unexplained, but it suggests that there may be underlying immunological mechanism that contribute the high incidence of miliary TB in this population.

This study is subject to some limitations. First, this research only collected data from Beijing Ditan Hospital rather than from multiple sites nationwide, which may limit the relevant scope of our study. Second, as a retrospective study, the prevalence of paradoxical TB-IRIS may be underestimated, as noted in a previous meta-analysis [17] Third, the immunocompromised conditions and comorbid diabetes and specially increase the risk for severe form of TB [26, 27]. However, considering that the HIV epidemic in China is attributed to men who have sex with men among young adults [28], the low incidence of comorbidities in the special population hinders the interpretation of correlation between TB-IRIS and underlying diseases. Finally, the patients enrolled in this study did not complete the follow-up interview. Thus we could not assess mortality associated with paradoxical TB-IRIS. Despite these limitations, our results firstly provide a snapshot of paradoxical TB-IRIS in Chinese population.

## Conclusion

In conclusion, this study describes the prevalence and risk factors of paradoxical TB-IRIS in China. Our data demonstrate that approximate one quarter of patients coinfected with TB and HIV develop paradoxical TB-IRIS after initial of ART therapy. Lower baseline CD4 count and rapid increase in CD4 count are the major risk factors associated with the occurrence of paradoxical TB-IRIS. In addition, patients younger than 25 years of age are most likely to have miliary TB among paradoxical TB-IRIS cases. Further studies are urgently needed to elucidate the molecular mechanism underlying pathophysiology of paradoxical TB-IRIS.

## Data Availability

The datasets generated and analyzed from the current study are not publicly available at this time as further analyses are ongoing, but are available from the corresponding author on reasonable request.

## Abbreviations

ART: Antiretroviral therapy
HIV: Human immunodeficiency virus
TB: Tuberculosis
TB-IRIS: TB-associated immune reconstitution inflammatory syndrome
